# Biocatalytic hydroxylation of *n*-butane with in situ cofactor regeneration at low temperature and under normal pressure

**DOI:** 10.3762/bjoc.8.20

**Published:** 2012-02-02

**Authors:** Svenja Staudt, Christina A Müller, Jan Marienhagen, Christian Böing, Stefan Buchholz, Ulrich Schwaneberg, Harald Gröger

**Affiliations:** 1 Department of Chemistry and Pharmacy, University of Erlangen-Nürnberg, Henkestr. 42, 91054 Erlangen, Germany; 2 Department of Biotechnology, RWTH Aachen University, Worringerweg 1, 52074 Aachen, Germany; 3 Evonik Oxeno GmbH, Paul-Baumann-Straße 1, 45772 Marl, Germany; 4 Evonik Degussa GmbH, Industrial Chemicals, Paul-Baumann-Straße 1, 45772 Marl, Germany; 5 Faculty of Chemistry, Bielefeld University, Universitätsstraße 25, 33615 Bielefeld, Germany

**Keywords:** biotransformations, cofactor regeneration, green chemistry, hydroxylation, P450-monooxygenase

## Abstract

The hydroxylation of *n*-alkanes, which proceeds in the presence of a P450-monooxygenase advantageously at temperatures significantly below room temperature, is described. In addition, an enzymatic hydroxylation of the “liquid gas” *n*-butane with in situ cofactor regeneration, which does not require high-pressure conditions, was developed. The resulting 2-butanol was obtained as the only regioisomer, at a product concentration of 0.16 g/L.

## Introduction

**Scheme 1:**
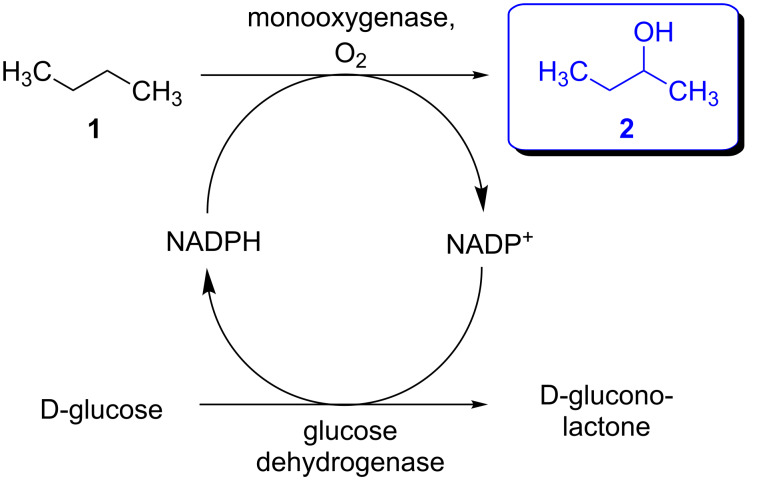
Biocatalytic *n*-butane hydroxylation with enzymatic in situ cofactor regeneration.

The (regioselective) oxidative functionalization of nonfunctionalized alkanes represents one of the most challenging types of reactions that organic chemists currently face [1–3]. A particular challenge in this field is the functionalization of volatile *n*-alkanes, which are present as gases under ambient conditions. A short *n*-alkane molecule that fulfils these criteria, of (i) being a gas at ambient temperature and pressure, and of (ii) being regioselectively hydroxylated, is *n*-butane. This compound is a so-called “liquid gas”, having a boiling point of −0.5 °C. A regioselective hydroxylation would yield either 1- or 2-butanol, which are bulk chemicals currently produced by chemocatalysis [4]. So far, reports on enzymatic functionalization of the liquid gas *n*-butane are rare, although some monohydroxylations were reported by various groups, leading to 1-butanol and 2-butanol with different product distributions [5–9]. When a methane monooxygenase from *Methylocystis* sp. was used, a ratio of 58:42 was obtained for the product isomers 1- and 2-butanol, respectively [7]. However, use of the hydroxylase unit only, in combination with hydrogen peroxide, furnished exclusively 2-butanol. A perfect regioselectivity of 100% for 2-butanol was reported by the group of Arnold using a P450-monooxygenase BM-3 mutant [8]. Recently, Reetz et al. reported a remarkable improvement when using a perfluoro carboxylic acid as an additive, thereby accelerating the hydroxylation as catalyzed by a P450-monooxygenase BM-3 mutant in combination with high pressure (10 bar) at 25 °C [9]. So far most hydroxylations of *n*-butane were performed without in situ cofactor regeneration, which is, however, a prerequisite for a synthetically useful process [10]. An exception is the recently reported process by Reetz et al. [9], which is based on the use of a glucose dehydrogenase and D-glucose as a cheap cosubstrate for in situ cofactor regeneration. Independently, in our work we focused on a biocatalytic hydroxylation of *n*-butane, which also proceeds with this type of in situ cofactor regeneration but does not require high-pressure conditions. In particular, we were interested in a practical experimental setup in which *n*-butane is utilized directly in liquid form (Scheme 1), and thus can be applied easily in laboratories without high-pressure equipment. In the following we report such a process.

For in situ cofactor regeneration in this regioselective hydroxylation of *n*-butane to 2-butanol in the absence of high-pressure conditions, the well-established glucose dehydrogenase/D-glucose cofactor regeneration system was chosen due to the successful use of this method on an industrial scale in several redox applications [11–12]. Since its low boiling point (−0.5 °C) represents a particular challenge for the preparative hydroxylation of *n*-butane, we became interested in the development of a biocatalytic hydroxylation that can be carried out at temperatures significantly below room temperature. This would limit the removal of *n*-butane into the gas phase, thus keeping the overall concentration of *n*-butane in the liquid phase at a reasonable level (at least over a certain period of time, which is dependent also on the volume of the gas phase).

## Results and Discussion

**Scheme 2:**
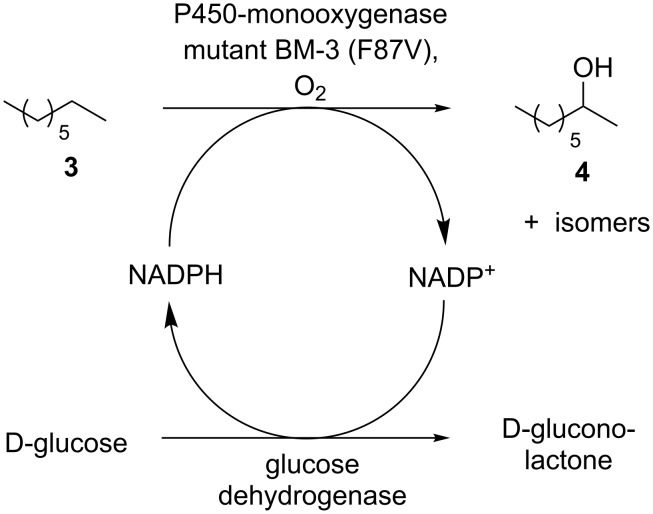
Biocatalytic *n*-octane hydroxylation with enzymatic in situ cofactor regeneration.

For our initial study of the ability of the P450-monooxygenase to exhibit sufficient activity even at a decreased reaction temperature, we used *n*-octane as a model substrate due to its higher boiling point and easier handling compared to *n*-butane. As a catalyst for the hydroxylation a P450-monooxygenase mutant of BM-3 (F87V) was used [13–16], and the in situ cofactor regeneration was conducted with a commercially available glucose dehydrogenase. This enzyme converts D-glucose into D-gluconolactone (which is then hydrolyzed in situ to D-gluconic acid), thus regenerating the reduced cofactor NADPH required for the hydroxylation step (Scheme 2).

As a benchmark reaction we carried out this transformation at room temperature (on 5 mL scale with 10 mol % of NADP^+^), forming 0.42 g/L of octanol isomers (Table 1, entry 1).

**Table 1:** Influence of reaction temperature and amount of cofactor on the enzymatic hydroxylation of *n*-octane.

Entry	NADP^+^ (mol %)	reaction temperature	product conc. (g/L)^a^

1	10	rt^b^	0.42
2	2	rt^b^	0.41
3	2	8 °C^c^	0.47

^a^Product concentration refers to 2-octanol and other octanol isomers; ^b^rt = room temperature for 24 h; ^c^8 °C for 8 h, then slow warm up to rt within the following 16 h.

The determination of the product concentration appeared to be a more reliable parameter to characterize the performance of the hydroxylation reaction, compared with the conversion due to evaporation of *n*-octane from the reaction mixture during the reaction. Using a decreased cofactor amount of 2 mol % gave a comparable performance resulting in 0.41 g/L of octane isomers (Table 1, entry 2). Notably, when starting this reaction at a decreased temperature of 8 °C an increased formation of octanol isomers of 0.47 g/L was observed (Table 1, entries 2 and 3). This clearly indicates that P450 BM-3-type monooxygenases operate suitably at low temperature, thus offering interesting perspectives for applications with liquid gases.

**Scheme 3:**
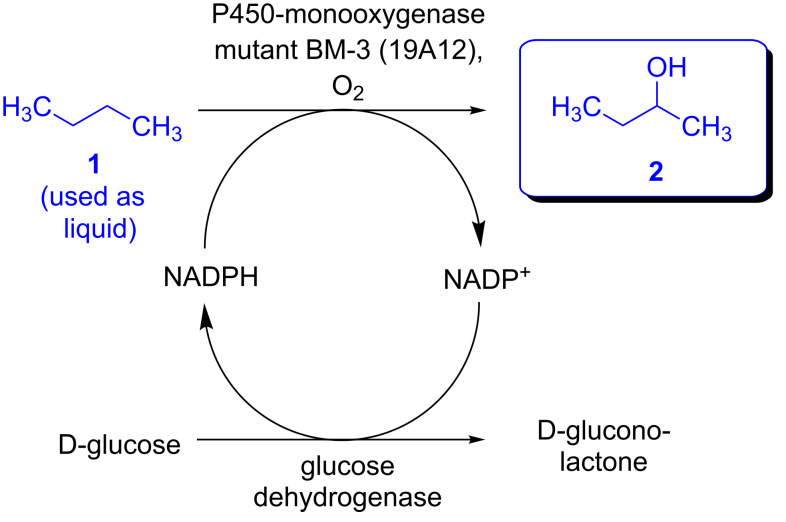
Enzymatic hydroxylation of *n*-butane (used as liquid) with in situ cofactor regeneration.

Encouraged by these results we then focused on the hydroxylation of the so-called “liquid gas” *n*-butane (boiling point: −0.5 °C), at a low starting temperature, which was chosen in the range of −5 to 8 °C. For the biotransformation of this liquid gas the following experimental procedure was carried out: First, all components required for the reaction (in a particular solvent) were cooled and then transferred into a precooled reaction flask. Subsequently, *n*-butane was added from a lecture bottle as the final component, and the reaction flask was sealed. The reaction mixture was kept at a given reaction temperature for 8 h, followed by a slow warm-up to room temperature within the following 16 h. In one experiment at 0 °C the work-up was directly carried out without a warm-up of the reaction mixture. As a P450-monooxygenase, the 19A12-mutant of the BM-3 enzyme was used, since this enzyme was reproted to be suitable for short-chain *n*-alkanes [8]. Once again, a glucose dehydrogenase from *Bacillus* sp. was used for enzymatic cofactor regeneration (according to the reaction concept shown in Scheme 1) in the presence of 0.09 mol % of NADP^+^ as a cofactor (Scheme 3).

When the reaction was conducted at room temperature, as a benchmark process, a product formation of 0.06 g/L of 2-butanol as the only regioisomer was observed (Table 2, entry 1).

**Table 2:** Enzymatic hydroxylation of *n*-butane.

Entry	reaction temperature	product conc. of **2** (g/L)^a^

1	rt^b^	0.06
2	8 °C^c^	0.10
3	0 °C^c^	0.16
4	0 °C^d^	0.14
5	−1/−2 °C^c^	0.13
6	−5 °C^c^	0.04

^a^Product concentration refers to 2-butanol as the only regioisomer observed; ^b^rt = room temperature for 24 h; ^c^this reaction temperature was maintained for 8 h, followed by a slow warm-up to rt within the following 16 h; ^d^0 °C for 8 h, then direct work-up.

Interestingly, we found a remarkable improvement in the product formation with concentrations of 0.10 g/L and 0.16 g/L achieved when lowering the initial reaction temperature significantly to 8 °C and 0 °C, respectively, for 8 h (Table 2, entries 2 and 3). These results, jointly with an experiment at 0 °C involving direct work-up without warm-up to room temperature (Table 2, entry 4), underline that the applied P450-monooxygenase mutant retains sufficient activity even at low reaction temperatures of 0 °C and 8 °C (Table 2, entries 2–4). When the reaction temperature was lowered further, to −1/−2 °C and −5 °C, less satisfactory results were obtained (Table 2, entries 5 and 6). An explanation for these lower product formations may be due to the highly viscous and frozen reaction mixtures that were obtained at −1/−2 °C and −5 °C. In order to make sure that this result was not in fact due to a loss of activity of the glucose dehydrogenase from *Bacillus* sp. at low temperatures, the activity of this enzyme was determined at −10 °C (40 vol % of glycerol) and at −5 °C (30 vol % of glycerol). In this study, the glucose dehydrogenase showed a high remaining activity of 81% at −10 °C and an activity of 82% at −5 °C after 24 h of stirring. Thus, the current optimized process for the desired hydroxylation of *n*-butane is based on a reaction temperature of 0 °C for 8 h, followed by a slow warm-up to room temperature (within the subsequent 16 h), which gives exclusively 2-butanol as the only regioisomer at a product concentration of 0.16 g/L (Table 2, entry 3).

## Conclusion

In conclusion, the use of P450-monooxygenases in the hydroxylation of *n*-alkanes, which proceeds advantageously at decreased reaction temperatures, has been reported. In addition, a biocatalytic oxidation of *n*-butane, as a representative example for a “liquid gas”, was carried out with enzymatic in situ cofactor regeneration and without the need for high-pressure conditions, in a highly regioselective fashion. This process, running at an initial reaction temperature of 0 °C, led exclusively to the formation of 2-butanol as the only regioisomer, at a product concentration of 0.16 g/L. The practical experimental setup of this process allows the direct use of *n*-butane, which has a boiling point of −0.5 °C, in liquid form and can be easily applied in laboratories lacking high-pressure equipment. A current challenge, addressed in our laboratories, is the further improvement of process efficiency and volumetric productivity of this enzymatic oxidation process. In addition, studies of enzyme stability during this biotransformation process are planned as future work.

## Experimental

### Bacterial strains and expression vectors

The expression strains used in this work are *E. coli* DH5α pCWori P450 BM-3 F87V [16] and *E. coli* BL21 Gold (DE3) lacI^Q1^ P450 BM-3 19A12 (this work).

#### Construction of the P450-monooxygenase BM-3 19A12 expression system

The heme domain of the P450-monooxygenase BM-3 19A12 variant [17] was purchased as synthetic DNA (Life Technologies, Darmstadt, Germany) and subcloned into the pET28a(+)-derived pALXtreme-1a vector, harbouring the wild-type P450 BM-3 gene from *Bacillus megaterium* (CYP102A1) [18]. For the purpose of replacing the wild-type heme domain of P450-monooxygenase BM-3, both the synthetic DNA and vector were cut for 1 h with 1 U each of FastDigest® endonucleases NcoI and MssI (Fermentas, St. Leon-Rot Germany) and purified by gel extraction (NucleoSpin^®^ Gel Clean up, Macherey-Nagel, Düren, Germany). A ligation reaction was performed with the T4 DNA Ligase (Fermentas, St. Leon-Rot Germany) according to the manufacturer’s instructions. The ligation reaction was subsequently transformed into chemically competent *E. coli* BL21 Gold (DE3) lacI^Q1^ cells [18], which were prepared by following a standard protocol [19]. For the chemically competent cells a transformation efficiency of 1 × 10^7^ cfu/µg pUC19 was determined.

#### Expression and isolation of the P450-monooxygenase BM-3 F87V and 19A12 variants

Both P450-monooxygenase BM-3 variants, P450-monooxygenase BM-3 F87V and P450-monooxygenase BM-3 19A12, were expressed in 2 L shaking flasks containing 400 mL TB expression media, which was supplemented with 400 µL trace elements (0.64 µM CuSO_4_, 0.69 µM ZnSO_4_, 0.72 µM MnSO_4_, 0.76 µM CoCl_2_, 59.43 µM Na_2_-EDTA, 61.79 µM FeCl_3_) and the respective antibiotic (for *E. coli* DH5α, harbouring pCWori, 100 µg/mL ampicillin and for *E. coli* BL21 Gold (DE3) LacI^Q1^, harbouring pALXtreme-1a, 50 µg/mL kanamycin). Cultivation, induction and expression were performed as previously described [20]. *E. coli* cells were harvested by centrifugation (10 min, 2900 g at 4 °C) and cell pellets were washed in 50 mM KP_i_ buffer (pH 7.0), and stored at −20 °C overnight. For preparation of crude cell extracts, frozen cell pellets were resuspended in 50 mM KP_i_ buffer (pH 7.0) prior to disruption with an Avestin EmulsiFlex-C3 high-pressure homogenizer (Ottawa, ON, Canada) by applying three cycles of 1500 bar. The lysate was centrifuged (30 min, 16000 g at 4 °C) in a Sorvall RC-6 Plus centrifuge (Thermo Scientific, Rockford, IL, USA) and further clarified by filtration through a 0.45 µm filter (Roth, Karlsruhe, Germany). Subsequently, crude cell extracts were shock-frozen in liquid nitrogen, lyophilized for 48 h at −54 °C in an Alpha 1-2 LD plus Freeze dryer (Christ, Osterode am Harz, Germany) and stored at −20 °C until further use.

### Typical procedure for the hydroxylation of *n*-octane with in situ cofactor regeneration (according to the reactions described in Table 1)

To 5 mL of phosphate buffer (pH 7.0, 50 mM) in a 10 mL flask, the F87V-mutant of P450-monooxygenase BM-3 (3.82 U; referring to the activity of *n*-octane), *n*-octane (16.3 µL; 0.1 mmol), D-glucose (90.1 mg; 0.5 mmol) and glucose dehydrogenase from *Bacillus* sp. (Amano Enzyme Inc.; 18 U referring to the activity for D-glucose) were added. After 20 min of stirring at rt or 8 °C, the reaction was started by the addition of 2 mol % (1.48 mg) or 10 mol % (7.44 mg) of NADP^+^. The mixture was stirred in a sealed flask for 24 h at rt, or for 8 h at 8 °C, followed by a slow warm-up to rt in the subsequent 16 h. Then, pyridine (0.3 mmol) was added as an external standard to the reaction mixture. For the work-up step the aqueous reaction mixture was subdivided into five portions. Each of them (1 mL) was filled into a 2 mL Eppendorf vial and extracted with methylene chloride (1 mL; by shaking the vials in a thermomixer at 25 °C for 30 min and removal of the aqueous layers with a syringe). The work-up step was repeated twice. In total, the aqueous layers were extracted three times with methylene chloride (3 × 5 mL). The organic and aqueous layers were separated by centrifugation for 30 min at 13.000 rpm. Afterwards the aqueous layers were removed by a syringe and the combined organic layers were evaporated at 900 mbar and 40 °C for about 30 min under vacuum. The amount of the formed product (in g of octanol isomers per L of reaction volume) was determined by ^1^H NMR spectroscopy and from the output weight of the crude product, or by ^1^H NMR spectroscopy in comparison with the quantity of the external standard.

### Typical procedure for the hydroxylation of *n*-butane with in situ cofactor regeneration (according to the reactions described in Table 2)

To 10 mL of cooled phosphate buffer (pH 7.0, 50 mM) in a cooled 100 mL flask, the 19A12-mutant of P450-monooxygenase BM-3 (3.82 U; referring to the activity of *n*-heptane), D-glucose (90.1 mg; 0.5 mmol), glucose dehydrogenase from *Bacillus* sp. (Amano Enzyme Inc.; 18 U referring to the activity for D-glucose) and NADP^+^ (1.48 mg; 0.002 mmol) were added. Immediately after *n*-butane was filled into the flask from a lecture bottle (entry 1: 163 mg; entry 2: 185 mg; entry 3: 175 mg; entry 4: 246 mg; entry 5: 323 mg; entry 6: 435 mg), the flask was sealed and the mixture was stirred in the sealed flask for 24 h at rt (entry 1), or for 8 h at a reaction temperature in the range of −5 °C to 8°C (entries 2–6), followed in most cases by a slow warm-up to rt in the subsequent 16 h (entries 2,3,5,6). The work-up was carried out as described above in the typical procedure for the hydroxylation of *n*-octane, with the exceptions that pyridine was not added and that the combined organic layers were evaporated at 900 mbar and 40 °C for about 40 min. The amount of formed product (in g of 2-butanol per L of reaction volume) was determined by ^1^H NMR spectroscopy and from the output weight of the crude product.
